# COLOMBOS: Access Port for Cross-Platform Bacterial Expression Compendia

**DOI:** 10.1371/journal.pone.0020938

**Published:** 2011-07-14

**Authors:** Kristof Engelen, Qiang Fu, Pieter Meysman, Aminael Sánchez-Rodríguez, Riet De Smet, Karen Lemmens, Ana Carolina Fierro, Kathleen Marchal

**Affiliations:** Department of Microbial and Molecular Systems, Katholieke Universiteit Leuven, Heverlee-Leuven, Belgium; Cairo University, Egypt

## Abstract

**Background:**

Microarrays are the main technology for large-scale transcriptional gene expression profiling, but the large bodies of data available in public databases are not useful due to the large heterogeneity. There are several initiatives that attempt to bundle these data into expression compendia, but such resources for bacterial organisms are scarce and limited to integration of experiments from the same platform or to indirect integration of per experiment analysis results.

**Methodology/Principal Findings:**

We have constructed comprehensive organism-specific cross-platform expression compendia for three bacterial model organisms (*Escherichia coli*, *Bacillus subtilis*, and *Salmonella enterica* serovar Typhimurium) together with an access portal, dubbed COLOMBOS, that not only provides easy access to the compendia, but also includes a suite of tools for exploring, analyzing, and visualizing the data within these compendia. It is freely available at http://bioi.biw.kuleuven.be/colombos. The compendia are unique in directly combining expression information from different microarray platforms and experiments, and we illustrate the potential benefits of this direct integration with a case study: extending the known regulon of the Fur transcription factor of *E. coli*. The compendia also incorporate extensive annotations for both genes and experimental conditions; these heterogeneous data are functionally integrated in the COLOMBOS analysis tools to interactively browse and query the compendia not only for specific genes or experiments, but also metabolic pathways, transcriptional regulation mechanisms, experimental conditions, biological processes, etc.

**Conclusions/Significance:**

We have created cross-platform expression compendia for several bacterial organisms and developed a complementary access port COLOMBOS, that also serves as a convenient expression analysis tool to extract useful biological information. This work is relevant to a large community of microbiologists by facilitating the use of publicly available microarray experiments to support their research.

## Introduction

Microarrays are the main technology for large-scale transcriptional gene expression profiling. Scientific journals generally require the deposit of these high-throughput experiments in public microarray databases, such as Gene Expression Omnibus (GEO) [1] or ArrayExpress [2], upon publication. These databases are an extremely rich source of information, containing freely accessible data for thousands of experiments and a multitude of different organisms, and in theory provide an opportunity to analyze gene expression of a particular species at a global level. They also hold the potential to expand the scope of any smaller scale study: mining the information contained in such databases offers molecular biologists the possibility to view their own dedicated experiments and analysis in light of what is already available. So far however, this wealth of public information remains largely untapped because these databases do not allow for a direct and integrated exploration of their data. The opportunity of combining all public experiments for a single organism has not been explored due to practical issues that can ultimately be attributed to the large heterogeneity inherent to microarray data. Data sets originate from different experimenters or labs and microarrays do not constitute a uniform technology. Multiple microarray platforms exist and are manufactured in different ways. Even for similar platforms, protocols for sample preparation, labeling, hybridization and scanning can vary greatly. There are also no requirements imposed [3], [4] regarding the format of the platform descriptions and expression measurements themselves, as well as the degree of preprocessing done on these values, which further complicates the matter of experiment integration from a practical point of view.

Despite such difficulties, several initiatives exist to actively build expression compendia from public resources. Most existing compendia can roughly be divided in two groups [5]: those that directly integrate single-platform experiments, and those that indirectly integrate cross-platform experiments. Combining data from a single platform makes the in-between experiment normalization and probe mapping relatively straightforward, so that the quantitative measures of gene expression can be analyzed directly across experiments. Most single-platform compendia databases, such as for instance M^3D^ [6], or the commercial Genevestigator [7], focus on Affymetrix, one of the more robust and reproducible platforms [8], [9]. Combining data from different platforms, even to the extent of combining data from single- and dual-channel microarrays, is generally done by indirect meta-analysis as opposed to directly integrating the actual expression values: one first applies the desired analysis procedure (e.g. identifying differentially expressed genes, clustering gene expression profiles, etc.) on each single data set within the compendium separately, and subsequently combines the derived results. These compendia are often topic-specific, collecting all publicly available experimental information related to a subject matter of interest. ITTACA [10] and ONCOMINE [11], for instance, focus on cancer in human; Gene Aging Nexus [12] on aging in several species. There are exceptions though, such as the large ATLAS [13] initiative from ArrayExpress,

Most of these compendia center on eukaryotic organisms; only M^3D^ has substantial compendia for two bacterial species (*Escherichia coli* and *Shewanella oneidensis*). The compendia in M^3D^ also have the advantage of retaining actual expression values, which broadens the scope of potential analysis procedures compared to indirect meta-analysis, but they are limited in the number of experiments they can include due to their single-platform nature. For eukaryotic model organisms considerable amounts of data are available and relying on only one platform can still lead to sizeable compendia with a broad scope in condition content, such as the human compendium constructed based on the Affymetrix U133A platform with over 5000 samples [14]. For prokaryote organisms, even model organisms such as *E. coli*, much less data is available and a significant portion is missed out on when considering only one platform. To have the advantage of direct integration, while not being limited to a single platform, we have devised a strategy that directly integrates expression data across platforms and experiments, and have used it to create expression compendia for several bacterial organisms. To increase their usability for a large community of microbiologists, these compendia have also been extensively annotated and are now being made available through COLOMBOS. COLOMBOS stands for *COLlection Of Microarrays for Bacterial OrganismS*. It is a web portal that provides easy access to the compendia and has an integrated suite of data tools for exploring, visualizing, and analyzing the expression data.

## Results and Discussion

### Database content

Currently COLOMBOS provides access to fully annotated public expression compendia for three bacterial model organisms: *Escherichia coli*, *Bacillus subtilis*, and *Salmonella enterica* serovar Typhimurium (see Table 1 for a detailed overview of their respective content). These expression compendia are essentially organism-specific matrices of expression values derived from publicly available microarray experiments which are homogenized to make them comparable. The rows of a compendium matrix correspond to the known genes of the organism in question. We refer to the columns as ‘condition contrasts’ because they do not represent single experimental conditions, but in fact always represent the difference between a test and reference condition (the expression values themselves are calculated as expression logratios). Converting absolute measures of expression into expression changes is the principal means for rendering expression values comparable across platforms and experiments. Relative expression calculated intra-experiment/platform (i.e. between two conditions measured for the same microarray experiment and platform) negates much of the platform and experiment specific variation that makes it impossible to reliably compare the absolute quantities reported in different experiments [15].

**Table 1:** An overview of the content of the three expression compendia that can be accessed through COLOMBOS.

	*Escherichia coli*	*Bacillus subtilis*	*Salmonella enterica* serovar Typhimurium
**Number of genes**	4295	4105	4525
**Number of contrasts**	1429	259	717
***source DB***	GEO, AE	GEO	GEO
***microarrays***	1483	265	723
***experiments***	84	9	25
***platforms***	35	13	9
**Missing values**	6.1%	6.40%	3.90%
**Condition properties**	242	67	77
**Condition ontology terms**	56	24	23
**External DBs**			
***pathway***	EcoCyc	BioCyc	BioCyc
***regulon***	RegulonDB	DBTBS	
***operon***	EcoCyc	BioCyc	BioCyc
***GO***	UniProt GOA	UniProt GOA	UniProt GOA

In order to be able to interpret and compare the expression logratios across an entire compendium, we have also extensively annotated all contrasts using a set of formal hierarchically-structured condition properties (representing for instance mutations, compounds in the growth medium, treatments, and general growth conditions). This contrast annotation is done to structure the large amounts of potentially useful information that remain untapped due to the non-standardized condition descriptions in public databases. The annotation is complemented with a condition ontology that groups the condition properties under one or more ontology terms. It serves as a higher level organization, and provides a biologically more intuitive view of the condition contrast annotation by assigning properties of seemingly distinct categories to the same biological process. For example, in our *Escherichia coli* compendium the condition ontology term ‘response to oxygen levels’ includes condition properties that are linked to cellular processes that are dependent on oxygen availability, such as *fnr* mutations (a global oxygen responsive transcriptional regulator), NO_2_ concentration (an electron transport decoupler), agitation of the growth medium, actual oxygen levels, etc. Apart from a thorough description of the represented biological conditions, we have also incorporated several sources of information from main curated databases (UniProt GOA [16], EcoCyc [17], BioCyc [18], RegulonDB [19], and DBTBS [20]) into each of the microbial compendia. This includes additional data regarding gene function and genomic organization, metabolic pathways, and transcriptional regulation mechanisms. Both the condition annotation and additional gene information are integrated into the COLOMBOS data analysis tools in a functional manner to interactively browse and query the compendia (see Methods). If users so desire however, they can also download the compendia in their entirety.

### Case study – Fur regulatory targets

In the following case study we illustrate the benefits of exploiting the direct integration of expression values, as well as the ease with which one can make interesting biological discoveries using the COLOMBOS data analysis tools (see Methods for a detailed description of their functionalities). A straightforward application provided by COLOMBOS is the ability to find genes which show similar expression behavior with a starting set of genes for relevant condition contrasts. Since co-expression might infer co-regulation, we can use this approach to obtain a list of potential target genes that might also be regulated by the same transcription factor. In this example, we will use COLOMBOS to identify novel potential targets for the Fur transcription factor of *Escherichia coli*. Fur mostly regulates genes related to iron homeostasis and is strongly conserved across many Gram-negative and Gram-positive bacteria [21]. It has received a lot of interest in the past for its role in iron-limited conditions, such as those encountered by pathogenic strains in their hosts [22]. Fur has mostly been reported as a direct repressor of its target genes, but is considered a dual regulator: activation occurs inderectly by transcriptional repression of a small antisense RNA RhyB [23]. Fur has also been known to mediate combinatorial responses along with many other transcription factors [24], [25]. In the latest release of RegulonDB [19], Fur is described as having 98 target sites in 43 distinct promoters, with 28 of these promoters known to be subject to combinatorial regulation. The results of all data analysis steps discussed here are available in the case study data set accessible from the COLOMBOS home page.

An initial set of 39 genes of the Fur regulon was constructed using the regulatory information integrated in COLOMBOS. Only genes known to be regulated by Fur alone, or by Fur in combination with the global regulators CRP, H-NS and/or FNR were selected. All other cases where known combinatorial regulation could occur were not included in the initial set because they might result in more complex, less homogenous transcriptional responses. For similar considerations, if the activating sigma factor was known, only genes responsive to the household σ^70^ were retained in the initial set. For this initial gene set the most relevant condition contrasts in the compendium were then selected, i.e. the contrasts where these genes showed the highest and most coherent response: a relevance cut-off (see Supplementary Text S1) of 1 resulted in 97 contrasts. Not all of the retained genes show a similar expression profile for the retained contrasts however, which might be attributed to unknown active forms of combinatorial regulation or the dual regulatory function of Fur. Since we wanted to continue with a set of strongly co-expressed genes, COLOMBOS was used to further clean the initial gene set by removing genes that had a correlation smaller then 0.8 with the mean of the initial set for the selected contrasts. Next we used COLOMBOS to extend the remaining set of 30 genes with additional ones that follow the same expression pattern for the selected contrasts (a correlation bigger than 0.8 was used as cut-off value), under the assumption that these constitute potential Fur targets. In this way, 19 extra genes were retrieved (Table 2), 7 of which were part of the Fur regulon but were not included in the initial set because they were known to be subject to regulation by additional transcription factors. The fact that these Fur-regulated genes were nevertheless retrieved might indicate that the additional combinatorial regulation was not active under the surveyed conditions.

**Table 2:** Finding potential novel Fur targets –a case study.

Locus tag	Name	Description	Operon	Known	COLOMBOS	Meta-analysis	Evidence
b1681	*sufD*	SufBCD Fe-S cluster scaffold	*sufABCDSE*	+		+	Fur, OxyR, IHF, lscR
b1683	*sufB*	SufBCD Fe-S cluster scaffold	*sufABCDSE*	+	+		Fur, OxyR, IHF, lscR
b2392	*mntH*	Manganese transport protein	*mntH*	+	+	+	Fur, MntR
b2673	*nrdH*	Glutaredoxin-like protein	*nrdHIEF*	+	+	+	Fur, NrdR
b2674	*nrdI*	Not annotated	*nrdHIEF*	+	+	+	Fur, NrdR
b2675	*nrdE*	Ribonucleoside-P_i_ reductase 2 α	*nrdHIEF*	+	+	+	Fur, NrdR
b2676	*nrdF*	Ribonucleoside- P_i_ reductase 2 β	*nrdHIEF*	+	+	+	Fur, NrdR
b4291	*fecA*	Fe^3+^ dicitrate transport protein	*fecABCDE*	+	+		Fur, CRP, PdhR
b0468	*ybaN*	Inner membrane protein	*ybaN*		+		Predicted
b0804	*ybiX*	PKHD-type hydroxylase	*ybiX*		+		Predicted; Fur dependent expression
b1018	*efeO*	UPF0409 protein	*efeUOB*		+		Predicted; functional in related strain
b1452	*yncE*	Uncharacterized protein	*yncE*		+	+	Fur dependent expression
b1494	*pqqL*	Probable zinc protease	*pqqL*		+		Potential operon *yddAB_pqqL*
b1495	*yddB*	Uncharacterized protein	*yddAB*		+		Predicted
b1705	*ydiE*	Not annotated	*ydiE*		+		Predicted; Fur dependent expression
b2211	*yojI*	ATP-binding ABC transporter	*yojI*		+		
b3070	*yqjH*	Uncharacterized protein	*yqjH*		+	+	Predicted
b3337	*bfd*	Bacterioferritin-associated ferredoxin	*bfd-bfr*		+		Indirect RhyB regulation
b3410	*feoC*	Ferrous iron transport protein C	*feoABC*		+		TU *feoABC* with *feoA* known target
b4366	*bglJ*	Transcriptional activator protein	*yjjQ-bglJ*		+		

Of the 12 novel genes, most showed a high likelihood of being Fur targets (Table 2). Six of these genes (*yqjH*, *ydiE*, *ybaN*, *yncE*, *yddB* and *ybiX*) were previously predicted to have a Fur target site in their transcription unit promoter by at least one of two independent studies [22], [26] (in case of *ybiX* as part of the proposed *fiu_ybiX* operon). Transcription of three of these (*ydiE*, *yncE* and *ybiX*) was moreover shown to be altered in a specific Fe^2+^-Fur-dependent manner [27] and while little is known with regard to their function, the *ybiX* gene encodes a protein similar to an iron-regulated hydroxylase-encoding gene from *Pseudomonas aeruginosa*, further supporting a role for Fur in its transcriptional regulation. *pqqL* presents an interesting case: it encodes for a putative zinc peptidase and is chromosomally situated directly downstream of the predicted Fur regulated *yddAB* operon. Using COLOMBOS to select the most relevant condition contrasts for the three genes *yddA*, *yddB*, and *pqqL* (see loadable case study data set) indeed shows that these genes are subject to tight co-expression, opening up the possibility of them being transcribed as a single transcription unit and putting *pqqL* under influence of the *yddA* promoter. The *feoC* gene is annotated as part of *feoABC* transcription unit as of the latest RegulonDB release (v6.8), which was not yet incorporated in COLOMBOS at the time of the analysis. This places it under the influence of the *feoA* promoter, which is a known Fur target. The *bfd* gene is clearly functionally related to Fur, being involved in iron storage and release, and has predicted binding sites in its promoter [21]. *bfd* is also the first gene in the *bfd_bfr* operon, *bfr* encoding for an iron storage protein that is at the very least indirectly regulated by Fur as it has been shown that the expression of this gene is repressed by a small RNA RhyB, which in turn is repressed by Fur [23]. The complex Fur dependent regulation of *bfd_bfr* is also apparent by diverging expression responses for some of the selected contrasts. In the *E. coli* K12 strain, the gene *efeO* is part of an operon that has been disrupted due to a frame shift mutation. However, a Fur binding site was recently predicted in the *efeU* promoter [26] and it has been shown in the related *E. coli* Nissle 1917 strain that expression of *efeUOB* increases in response to iron-depleted conditions in a Fe^2+^-Fur-dependent manner [28].

COLOMBOS also provides the functionality to retrieve anti-correlated genes, which can be interesting to investigate the potential of dual regulation (activation or repression by the same regulator). In the case of our Fur module, none of the anti-correlated genes pass the threshold of −0.8, but it is interesting to note that the second best ranked gene (correlation −0.74) is *ftnA*. This gene was not yet assigned as a Fur target in the Regulon DB release included in COLOMBOS, but it was recently shown that *ftnA* is transcriptionally activated by Fur directly (as opposed to inderectly through RhyB as is usually the case for Fur mediated activation) by reversal of H-NS silencing [29].

While the retrieval of already known Fur regulon genes combined with a set of likely targets confirms that a careful co-expression analysis can lead to the identification of novel targets, this does not imply that the direct integration of expression data itself, as in our compendia, provides any benefits. To illustrate the advantage of using cross-platform compendia, we repeated the analysis on a per experiment basis (a ‘meta-analysis’ of 7 experiments from which the 97 contrasts above were selected). Note that, to maximize the quality of the results of this meta-analysis, we did not use all contrasts within each experiment, but only the most relevant ones (selected with the same relevance cut-off as before), and that we ignored experiments with two contrasts or less. When extending the initial 30 genes with the same correlation cut-off of 0.8, the number of additional genes for each experiment ranges between 389 and 1385, the union adding up to a total of 3361. Most of these genes are false-positives with respect to being members of the Fur regulon: within single experiments generally only a limited number of similar conditions are surveyed and this increases the chance of finding genes with similar up and down regulation patterns but not sharing the exact same regulatory program. Trying to counter this effect by increasing the correlation cut-off does not necessarily yield better results, a cut-off of 0.9 resulting in the union containing 2135 additional genes, one of 0.95 in 1361 genes. Therefore we retained only the intersection, i.e. those genes that were added by each of the per experiment extensions with a correlation cut-off of 0.8. This intersection constituted 8 additional genes (a cut-off of 0.9 resulted in only 4 added genes, 0.95 resulted in none), 6 of them already known Fur targets, and only two uncharacterized genes representing potential novel targets. All of these were also retrieved by the COLOMBOS cross-platform analysis, with the exception of a single already known Fur target, *sufD*. However, another gene of the *sufABCDSE* operon was selected by the cross-platform analysis (*sufB*; all other genes of the operon showed correlations with the initial set of just under 0.8), retrieving the same promoter as a Fur target.

### Conclusions and future directions

In this work we aim at closing the gap towards an encompassing expression resource for prokaryotic organisms and facilitate the use of information in publicly available microarray experiments for a large community of microbiologists. We have created fully annotated cross-platform expression compendia for three bacterial model organisms: namely *Escherichia coli*, *Bacillus subtilis*, and *Salmonella enterica* serovar Typhimurium. These compendia can be accessed through a web portal called COLOMBOS which also provides a suite of integrated analysis and visualization tools. To our knowledge, COLOMBOS is unique in offering compendia for *B. subtilis* and *S.* Typhimurium, and its *E. coli* compendium is the largest currently available. To maximally exploit the available expression data, several aspects of both compendia construction, as well as design and implementation of the analysis tools, are exclusive to COLOMBOS (see Table 3 for a conceptual comparison with similar initiatives). Most notably, the compendia were created by directly integrating expression measurements from different experiments and microarray platforms. The reputed low reproducibility between microarray experiments and platforms [8], [30] (although more promising findings have also been reported [15], [31], [32]) is not a legitimate argument for not combining them: short of an objective basis to dismiss certain measurements, a lack of agreement between two experiments does not render either invalid and might in fact be a strong motivation to integrate them. In our previous research directly combining expression data from different sources proved a valuable asset for reconstructing transcriptional networks [33], [34], [35], and here we wanted to take the principle of direct cross-platform integration to a higher level by generating large scale expression compendia with a broad applicability for biological discovery. Directly integrating expression data enables one to simultaneously assess multiple diverse conditions, relevant to the biological problem of interest and ensures a finer-grained view of condition dependent transcription responses that can lead to higher quality predictions, such as in the case study above for extending the known regulon of a transcription factor.

**Table 3:** Conceptual comparison of COLOMBOS with similar initiatives.

	COLOMBOS	M3D	GXA	GeneVestigator
**DB CONTENT**				
**Expression data** 1	Cross-platform compendia	Single platform compendia (Affymetrix)	Experiment centered (ArrayExpress meta-analysis)	Single platform compendia (Affymetrix)
**Organisms**	Prokaryotes (3)	Prokaryotes (2) and a eukaryote	Eukaryotes (10)	Eukaryotes (9) and a prokaryote
**Gene annotation**	Incroporation of multiple species-specific DBs	Referal to BioCyc, SGD	EBI	None
**Microarray annotation**	Microarray annotation and condition ontology^2^	Microarray annotation	Microarray annotation and condition ontology^2^	Microarray annotation
**Tools suite**	Interactive visualization, expression analysis	Visualization, expression analysis	Interactive visualization, expression analysis	Interactive visualization, expression analysis
**FUNCTIONALITY**				
**Expression analysis**	Multiple queries^3^	Single query	Single query	Single query (limited)
**Query genes by…**	Gene IDs; functional or structural characteristics	Gene IDs	Gene/protein IDs	Gene IDs
**Query arrays by…**	Experiment, annotation, or ontology	Experiment, annotation	Experiment, annotation, or ontology	Annotation
**Download**	Analysis results and/or entire compendia	Analysis results and/or entire compendia	Only experiments indirectly (through ArrayExpress)	Analysis results (limited)

1 Compendium: a data matrix (genes in rows, microarrays in columns) combining expression measurements from different experiments (an experiment being a set of microarrays submitted to the public DBs as such, implying that they were performed by the same lab and on the same technological platform). Single- vs. cross-platform: combining data from the same technological platform is relatively easy as the same preprocessing methodology can be employed; COLOMBOS is unique in combining data from different platforms using a specialized homogenization pipeline. Meta-analysis: expression data are not combined directly but experiments are analyzed separately where after the results are compared.

2 The biological conditions measured on a microarray are described with a set of formal terms which are organized into a higher level ontology. Such an ontology facilitates querying for related experiments or conditions.

3 Single versus multiple queries: query results can be retained in the COLOMBOS user workspace where they can be organized and structured, into larger ‘analysis projects’. This allows for integrative across-query analysis where relations between single query results can be explored, e.g. by combining or differentiating single query results.

We have also taken great care to provide an extensive formal condition contrast annotation and associated higher level condition ontology for all compendia. Microarray experiments that are committed to a public database, such as ArrayExpress or GEO, are required to comply to the MIAME standards [3], [4]. And while much effort has been taken to standardize the description of the experimental protocols used in a microarray experiment, there are no specifications of the format in which the surveyed biological conditions should be presented. The resulting cryptic, non-standardized condition descriptions in public databases do not enable computational comparison and automatic organizing of experiments which our annotation does. Another feat in which COLOMBOS is unique: this condition annotation is functionally integrated in the data analysis tools allowing the user to interactively browse and query the compendia, not only for specific arrays or experiments, but also for specific experimental conditions and biological processes. In a similar fashion, information from main curated microbial databases is also integrated to interactively browse and query the compendia for specific genes, pathways, transcriptional regulation mechanisms, and more.

Downloadable versions of the entire annotated compendia, as well as the COLOMBOS data analysis tools, are available at http://bioi.biw.kuleuven.be/colombos. In a half-yearly fashion new revisions of the compendia, updated with additional experiments, will be made available. We also plan to increase the current scope of organisms by adding new compendia for other bacterial species using a flexible framework for creating and updating cross-platform compendia which is currently in development. The data analysis tools incorporated in COLOMBOS will continue to be developed to offer users enhanced tools for analyzing and visualizing the compendia's expression data.

## Methods

### Cross-platform expression compendia

The compendia are built in three major steps. The first step is the retrieval of microarray experiments and associated platforms from Gene Expression Omnibus (GEO) and ArrayExpress. Representation discrepancies prevalent in experimental data directly obtained from online databases are systematically removed and the resulting data are then stored as available in a uniform format. ‘As available’ does not necessarily equate to raw scanner output, since there are no MIAME reporting standards regarding the measurement units of expression [3], [4]. Often raw intensities are not provided in the public databases (especially for older experiments), and only already processed data are reported. At this stage probes are also mapped in a platform-specific manner to a unique list of genes which is constructed based on the organism's RefSeq file at NCBI [36] and which corresponds to the rows of the final compendium. If probe sequences are available or can be obtained from the platform description, the mapping is driven by sequence homology searches using BLAST [37]. If not, a probe's target gene is identified by other probe info, namely -and in order of preference: locus tags, alternative gene tags, or common gene names.

In a next phase, the condition contrasts that will be represented in the compendium are defined and annotated. Based on their biological role in an experimental survey, hybridizations are labeled ‘reference’ or ‘test’ on a per experiment-and-platform combination basis and matched to produce a set of condition contrasts. For a single channel experiment, one or more hybridizations are chosen as references for the remaining tests. For dual channel experiments, usually one of every two array hybridizations serves as a reference to the other, as this inherently counters much probe spot associated variation in the measurements. There are exceptions however, such as when one of the hybridizations on an array does not constitute an identifiable and unique biological condition for which the transcriptome was assessed (e.g. a sample of genomic DNA or a pool of different samples that cannot be considered as biological replicates). These hybridizations are discarded and the experiment is further treated as if it was a single channel experiment. In this way we ensure that every contrast has a biologically interpretable meaning: its associated logratios measure changes in expression in response to quantifiable stimuli that are altered from reference to test. Using a set of formal hierarchically structured condition properties (representing for instance mutations, compounds in the growth medium, treatments, and general growth conditions), we can then specify the annotation of each condition contrast rigidly as a vector representing the differences for these property values between the test and reference condition. This representation enables a mathematical comparison and automatic organization of contrasts based on the conditions that are surveyed, but it is a labor intensive manual curation process where information often needs to be retrieved from original publications, supplementary data and occasionally directly from the authors. The condition properties themselves are further structured in a condition ontology tree. This ontology employs the same classes as the Gene Ontology biological process subtree terms [38] and maps the condition properties used to annotate the condition contrasts to one or more biological processes or functionalities they most likely affect.

The final part in the creation of a compendium is the homogenization of the expression data: several preprocessing procedures are conducted to render expression levels comparable between different experiments and platforms. Crucial steps in this preprocessing are array-specific and depend on both the technological platform that was used to perform the experiment, as well as on the reported units of expression and the type of normalizations that might have already been done. In general we adhere to the following principles: 1) whenever possible, raw intensities are preferred as data source over normalized data provided by the public repository, 2) no local background or mismatch probe correction procedures are performed to avoid an increase in intensity error variance for lower, less reliable intensity levels [39], [40], [41], 3) non-linear normalization techniques are performed to account for global inter-hybridization differences (e.g. loess fit to remove dye-related discrepancies on dual channel arrays [42], quantile normalization for high-density oligonucleotide experiments [43]) and 4) logratios are created for single-channel data according to the condition contrast definitions and combined with the dual channel measurements.

### COLOMBOS data analysis tools

**Figure 1:**
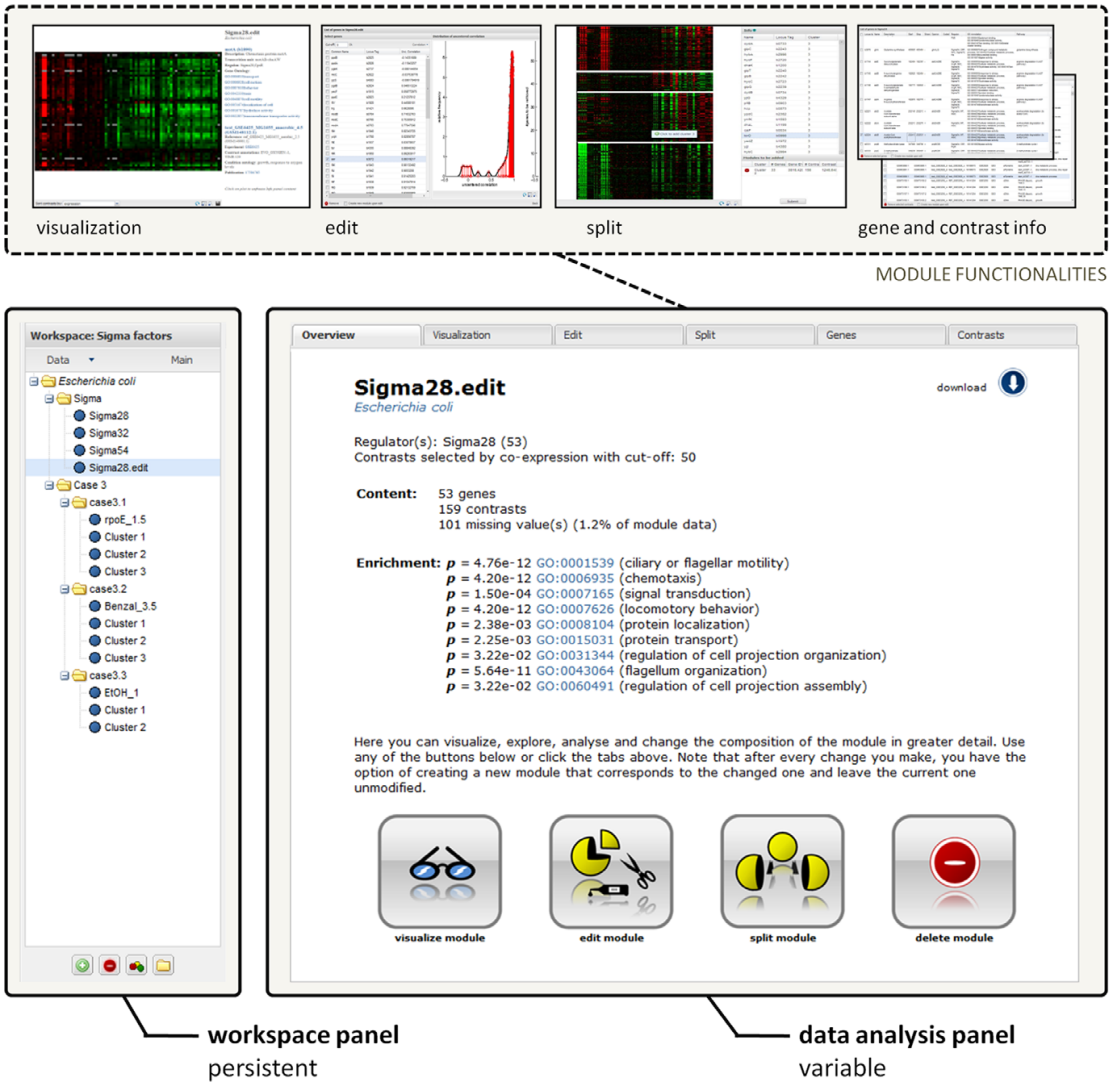
Screenshots of COLOMBOS data analysis components. The bottom part shows the two main panels of the data analysis page. The left hand workspace panel is always visible, containing an overview of the modules and the main analysis controls. The content of the right hand data analysis panel depends on the actions of the user. In this case it shows the overview page for a module selected in the workspace. This overview page not only provides some general information on the selected module, but also serves as a guide for further examination and analysis steps. These are illustrated at the top part of the figure and include visualization, content editing (demonstrated is the removal of genes based on expression profile similarity), splitting the module based on expression values (shown here in the gene direction), and exploration of gene and contrast information.

COLOMBOS also provides a suite of intuitive tools for exploring, visualizing, and analyzing the expression data in the compendia. The interface is divided in two main sections: a ‘Workspace panel’ to the left and a ‘Data analysis panel’ to the right (Figure 1). The workspace panel is always visible: it contains the main control elements and shows an overview of the data (the ‘workspace’) the user is working with. The right hand data analysis panel is where querying of the database and visualization and analysis of the expression data takes place.

All steps and procedures in the COLOMBOS analysis tools act on what we call expression ‘modules’. A module in COLOMBOS can be considered as a result of a single query to the database and is always a combination of a set of genes and a set of contrasts with corresponding expression values. Modules are dynamic in that at any time after creation their content can be altered by the user in various ways. In addition, multiple modules can be retained and organized in the workspace and can be analyzed simultaneously. As the basic *modus operandi*, modules create a general framework through which various interesting, but conceptually different biological questions can be handled.

Three different options are given for creating a module: by manually selecting only genes and have COLOMBOS automatically identify relevant condition contrasts, by manually selecting only condition contrasts and have COLOMBOS automatically identify sets of co-expressed genes, or by explicitly selecting both genes and condition contrasts manually. Depending on the gene annotations that are available for the selected organism in the public databases that COLOMBOS integrates (see Table 1), the set of genes can be selected as anything from an operon or a regulon, to enzymes representing a metabolic pathway, or any custom list of genes that one is interested in. Similarly, the module contrasts represent the biological conditions of interest and can also be retrieved in various ways, such as by experiment, by contrast annotation, or by condition ontology. When specifying only a set of genes, COLOMBOS will identify relevant condition contrasts based on the expression values of the selected genes in the compendium (user defined relevance cut-off that prioritizes both the magnitude as well as the consistency of the expression changes; see Supplementary Text S1 for more details). Starting from only condition contrasts, COLOMBOS retrieves the most variable genes for the defined contrasts and (as an optional step) can identify clusters of co-expressed genes within this selection, which can be added as distinct modules.

Once a module is defined, it can be visualized in an interactive manner (with the option to export high-quality images), its expression values and contrast annotation can be downloaded, it can be split up in multiple modules in either the gene or contrast direction by clustering the expression profiles, or it can be further edited in gene and/or contrast composition by using available gene and contrast annotations or by analysis of the expression values in the compendium. These functionalities of the analysis tools are illustrated in Figure 1, showing the overview page for a single module. The module overview page gives some basic module information (such as the number of included genes and contrasts, the number of missing values, and a list of Gene Ontology enrichment scores) and serves as a helping guide to further analyze and visualize the module's composition.

When multiple modules have been created, they can also be explored and edited together. Any number of modules can be collectively visualized (to explore potential overlap), can be merged into a new module, and can be subtracted from one another in gene or contrast content. Visually exploring the module overlap, both in gene and contrast composition, can serve as an important guide for deciding which modules may be grouped or subtracted.

Note that all of COLOMBOS' calculations, in both creating and editing modules, explicitly take into account the relative nature of the expression values by recognizing 0, implying no change, as the natural reference state of a logratio (for details see Supplementary Text S1). Gene profile similarities are calculated by default as the uncentered Pearson correlation, which assumes that the sample means (i.e. the means of two gene expression profiles across a set of condition contrasts) are zero. Standard deviations of gene profiles are calculated in a similar way (as the root of the mean sum of squared logratios).

## Supporting Information

Text S1 Scores used to edit and create modules based on expression values. COLOMBOS provides rich functionalities to create and/or edit expression ‘modules’, some of which are based on the expression values themselves. The calculations used in these procedures to score relevance of a contrast for a set of genes, similarity of genes across a set of contrasts, or variability of a gene across a set of contrasts, are explained in this supplementary.
(DOCX)

## References

[1] Barrett T, Troup DB, Wilhite SE, Ledoux P, Rudnev D (2009). NCBI GEO: archive for high-throughput functional genomic data.. Nucleic Acids Res.

[2] Parkinson H, Kapushesky M, Kolesnikov N, Rustici G, Shojatalab M (2009). ArrayExpress update–from an archive of functional genomics experiments to the atlas of gene expression.. Nucleic Acids Res.

[3] Brazma A (2009). Minimum Information About a Microarray Experiment (MIAME)–successes, failures, challenges.. Scientific World Journal.

[4] Brazma A, Hingamp P, Quackenbush J, Sherlock G, Spellman P (2001). Minimum information about a microarray experiment (MIAME)-toward standards for microarray data.. Nat Genet.

[5] Fierro AC, Vandenbussche F, Engelen K, Van de Peer Y, Marchal K (2008). Meta Analysis of Gene Expression Data within and Across Species.. Curr Genomics.

[6] Faith JJ, Driscoll ME, Fusaro VA, Cosgrove EJ, Hayete B (2008). Many Microbe Microarrays Database: uniformly normalized Affymetrix compendia with structured experimental metadata.. Nucleic Acids Res.

[7] Hruz T, Laule O, Szabo G, Wessendorp F, Bleuler S (2008). Genevestigator v3: a reference expression database for the meta-analysis of transcriptomes.. Adv Bioinformatics.

[8] Bammler T, Beyer RP, Bhattacharya S, Boorman GA, Boyles A (2005). Standardizing global gene expression analysis between laboratories and across platforms.. Nat Methods.

[9] Irizarry RA, Warren D, Spencer F, Kim IF, Biswal S (2005). Multiple-laboratory comparison of microarray platforms.. Nat Methods.

[10] Elfilali A, Lair S, Verbeke C, La Rosa P, Radvanyi F (2006). ITTACA: a new database for integrated tumor transcriptome array and clinical data analysis.. Nucleic Acids Res.

[11] Rhodes DR, Kalyana-Sundaram S, Mahavisno V, Varambally R, Yu J (2007). Oncomine 3.0: genes, pathways, and networks in a collection of 18,000 cancer gene expression profiles.. Neoplasia.

[12] Pan F, Chiu CH, Pulapura S, Mehan MR, Nunez-Iglesias J (2007). Gene Aging Nexus: a web database and data mining platform for microarray data on aging.. Nucleic Acids Res.

[13] Kapushesky M, Emam I, Holloway E, Kurnosov P, Zorin A (2010). Gene expression atlas at the European bioinformatics institute.. Nucleic Acids Res.

[14] Lukk M, Kapushesky M, Nikkila J, Parkinson H, Goncalves A (2010). A global map of human gene expression.. Nat Biotechnol.

[15] Shi L, Reid LH, Jones WD, Shippy R, Warrington JA (2006). The MicroArray Quality Control (MAQC) project shows inter- and intraplatform reproducibility of gene expression measurements.. Nat Biotechnol.

[16] Camon E, Magrane M, Barrell D, Lee V, Dimmer E (2004). The Gene Ontology Annotation (GOA) Database: sharing knowledge in Uniprot with Gene Ontology.. Nucleic Acids Res.

[17] Keseler IM, Bonavides-Martinez C, Collado-Vides J, Gama-Castro S, Gunsalus RP (2009). EcoCyc: a comprehensive view of Escherichia coli biology.. Nucleic Acids Res.

[18] Caspi R, Altman T, Dale JM, Dreher K, Fulcher CA (2010). The MetaCyc database of metabolic pathways and enzymes and the BioCyc collection of pathway/genome databases.. Nucleic Acids Res.

[19] Gama-Castro S, Jimenez-Jacinto V, Peralta-Gil M, Santos-Zavaleta A, Penaloza-Spinola MI (2008). RegulonDB (version 6.0): gene regulation model of Escherichia coli K-12 beyond transcription, active (experimental) annotated promoters and Textpresso navigation.. Nucleic Acids Res.

[20] Sierro N, Makita Y, de Hoon M, Nakai K (2008). DBTBS: a database of transcriptional regulation in Bacillus subtilis containing upstream intergenic conservation information.. Nucleic Acids Res.

[21] Chen Z, Lewis KA, Shultzaberger RK, Lyakhov IG, Zheng M (2007). Discovery of Fur binding site clusters in Escherichia coli by information theory models.. Nucleic Acids Res.

[22] Panina EM, Mironov AA, Gelfand MS (2001). Comparative analysis of FUR regulons in gamma-proteobacteria.. Nucleic Acids Res.

[23] Masse E, Gottesman S (2002). A small RNA regulates the expression of genes involved in iron metabolism in Escherichia coli.. Proc Natl Acad Sci U S A.

[24] Patzer SI, Hantke K (2001). Dual repression by Fe(2+)-Fur and Mn(2+)-MntR of the mntH gene, encoding an NRAMP-like Mn(2+) transporter in Escherichia coli.. J Bacteriol.

[25] Zhang Z, Gosset G, Barabote R, Gonzalez CS, Cuevas WA (2005). Functional interactions between the carbon and iron utilization regulators, Crp and Fur, in Escherichia coli.. J Bacteriol.

[26] Meysman P, Dang TH, Laukens K, De Smet R, Wu Y (2010). Use of structural DNA properties for the prediction of transcription-factor binding sites in Escherichia coli.. Nucleic Acids Res.

[27] McHugh JP, Rodriguez-Quinones F, Abdul-Tehrani H, Svistunenko DA, Poole RK (2003). Global iron-dependent gene regulation in Escherichia coli. A new mechanism for iron homeostasis.. J Biol Chem.

[28] Grosse C, Scherer J, Koch D, Otto M, Taudte N (2006). A new ferrous iron-uptake transporter, EfeU (YcdN), from Escherichia coli.. Mol Microbiol.

[29] Nandal A, Huggins CC, Woodhall MR, McHugh J, Rodriguez-Quinones F (2010). Induction of the ferritin gene (ftnA) of Escherichia coli by Fe(2+)-Fur is mediated by reversal of H-NS silencing and is RyhB independent.. Mol Microbiol.

[30] Tan PK, Downey TJ, Spitznagel EL, Xu P, Fu D (2003). Evaluation of gene expression measurements from commercial microarray platforms.. Nucleic Acids Res.

[31] Kuo WP, Liu F, Trimarchi J, Punzo C, Lombardi M (2006). A sequence-oriented comparison of gene expression measurements across different hybridization-based technologies.. Nat Biotechnol.

[32] Shi L, Tong W, Fang H, Scherf U, Han J (2005). Cross-platform comparability of microarray technology: intra-platform consistency and appropriate data analysis procedures are essential.. BMC Bioinformatics.

[33] Lemmens K, De Bie T, Dhollander T, De Keersmaecker SC, Thijs IM (2009). DISTILLER: a data integration framework to reveal condition dependency of complex regulons in Escherichia coli.. Genome Biol.

[34] Fadda A, Fierro AC, Lemmens K, Monsieurs P, Engelen K (2009). Inferring the transcriptional network of Bacillus subtilis.. Mol Biosyst.

[35] Zarrineh P, Fierro AC, Sánchez-Rodríguez A, De Moor B, Engelen K (2010). COMODO: an adaptive coclustering strategy to identify conserved coexpression modules between organisms.. Nucleic Acids Res.

[36] Pruitt KD, Tatusova T, Maglott DR (2007). NCBI reference sequences (RefSeq): a curated non-redundant sequence database of genomes, transcripts and proteins.. Nucleic Acids Res.

[37] Altschul SF, Madden TL, Schaffer AA, Zhang J, Zhang Z (1997). Gapped BLAST and PSI-BLAST: a new generation of protein database search programs.. Nucleic Acids Res.

[38] Gene Ontology Consortium (2010). The Gene Ontology in 2010: extensions and refinements.. Nucleic Acids Res.

[39] Ritchie ME, Silver J, Oshlack A, Holmes M, Diyagama D (2007). A comparison of background correction methods for two-colour microarrays.. Bioinformatics.

[40] Engelen K, Naudts B, De Moor B, Marchal K (2006). A calibration method for estimating absolute expression levels from microarray data.. Bioinformatics.

[41] Li C, Wong WH (2001). Model-based analysis of oligonucleotide arrays: expression index computation and outlier detection.. Proc Natl Acad Sci U S A.

[42] Yang YH, Dudoit S, Luu P, Lin DM, Peng V (2002). Normalization for cDNA microarray data: a robust composite method addressing single and multiple slide systematic variation.. Nucleic Acids Res.

[43] Bolstad BM, Irizarry RA, Astrand M, Speed TP (2003). A comparison of normalization methods for high density oligonucleotide array data based on variance and bias.. Bioinformatics.

