# Supplementary material for: COLOMBOS: Access Port for Cross-Platform Bacterial Expression Compendia
Source: PLoS One. 2011 Jul 14;6(7):e20938. doi: 10.1371/journal.pone.0020938 (PMC3136457; doi:10.1371/journal.pone.0020938)
Supplement: Text S1 — Scores used to edit and create modules based on expression values. COLOMBOS provides rich functionalities to create and/or edit expression ‘modules’, some of which are based on the expression values themselves. The calculations used in these procedures to score relevance of a contrast for a set of genes, similarity of genes across a set of contrasts, or variability of a gene across a set of contrasts, are explained in this supplementary. (DOCX) [file pone.0020938.s001.docx]

## Supplementary Text S1 – scores used to edit and create modules based on expression values

COLOMBOS provides rich functionalities to create and/or edit expression 'modules', some of which are based on the expression values themselves. The calculations used in these procedures to score relevance of a contrast for a set of genes, similarity of genes across a set of contrasts, or variability of a gene across a set of contrasts, are explained in this supplementary.

***Contrast relevance scores***

The default relevance score *c* of a condition contrast for a group of genes is calculated as the absolute inverse coefficient of variation of those genes’ expression values in this contrast. It is defined as the absolute mean divided by the standard deviation of the genes’ expression values:

$c= \frac{|\mu|}{\sigma}$

On the one hand, for expression values of the same mean, the higher the score, the less sparse the values are. It prioritizes the contrasts where genes’ expression values are more consistent. On the other hand, for expression values of the same standard deviation, the higher the score, the higher the mean. It prefers the contrasts where genes are highly expressed. The score thus serves as a measure that values both magnitude of expression change in response to a condition contrast, as well as coherence of expression within that contrast. The score identifies the most relevant contrasts as those where the genes 'act as one', showing the same, preferentially large, magnitude of expression change with individual variations ideally only constituting random Gaussian noise. From this notion, the score represents the number of standard deviations the mean expression value of this distribution is situated away from 0, and can be interpreted as a *Z*-score for the selected genes' expression change as a whole. (Note that in case of only one gene, the score of a condition contrast is degraded to the absolute expression value of that gene under it.)

We have also provided an alternative measure for the contrast relevance (selectable by the box in the top right of the contrast selection window) called 'M value cutoff'. The score assigned to the contrasts in this case is the minimum M value (i.e. the logratio) for the considered genes in case all genes' M values are positive, or the maximum absolute M value in case all genes' M values are negative. In case of both positive and negative M values exist for the considered genes, the contrast gets a score of 0.

***Gene similarity score***

The default similarity between a gene and a module's mean profile, is the *Uncentered Pearson’s correlation* calculated based on the formula:

where $\sigma_{x}^{(0)}=\sqrt{\frac{1}{n}\sum_{i=1}^{n} x_{i}^{2}}$; and $\sigma_{y}^{(0)}=\sqrt{\frac{1}{n}\sum_{i=1}^{n} y_{i}^{2}}$. Here represents the candidate gene expression data at condition contrast $i$, whereasrepresents a module’s mean expression value at contrast $i$. $\sigma_{x}^{(0)}$ and $\sigma_{y}^{(0)}$are both uncentered standard deviations assuming zero mean of the population, hence they are marked with superscript ‘(0)’. The higher the, the more similar the expression profile of a gene is to a module’s mean expression profile.

We have also provided an alternative measure for the gene similarity (selectable by the box in the top right of the contrast selection window) that calculates an uncentered version of the Spearman rank correlation. The Spearman rank correlation is calculated in the same way as the Pearson correlation but on the ranks of the data instead of the data itself. To calculate an uncentered version, instead of ranking all values from low to high, the positive logratios are ranked from low to high while the negative logratios are ranked from high to low and then assigned a negative sign; the mean rank is assumed 0. This uncentered Spearman rank correlation, compared to the uncentered Pearson correlation, has the advantage of being able to capture non-linear similarities, but the disadvantage of ignoring the actual magnitudes of expression changes and their distributions.

For ranking genes, there are three options provided based on the uncentered (rank) correlation score calculated. First one is ‘positive’ which uses directly as final score. The second option ‘absolute’ takes. It ranks both correlated and anti-correlated genes based solely on their similarities. Instead, the third option ‘negative’ takes as a score to favor only the anti-correlated genes.

***Gene variability***

The expression variability of a gene *x* for conditions *i*=1,…,*n* is calculated as the uncentered standard deviation:

$$\sigma_{x}^{(0)}=\sqrt{\frac{1}{n}\sum_{i=1}^{n} x_{i}^{2}}$$
